# Serum anti-Müllerian hormone (AMH) concentration during pregnancy: a longitudinal study

**DOI:** 10.1530/RAF-22-0128

**Published:** 2023-04-19

**Authors:** Sarah McCredie, Belinda An, Monika McShane, William Ledger, Christos A Venetis

**Affiliations:** 1 School of Women’s and Children’s Health, UNSW Medicine, UNSW, New South Wales, Australia; 2 Royal Hospital for Women, Randwick, New South Wales, Australia

**Keywords:** anti-Müllerian hormone, pregnancy, AMH, ovarian reserve

## Abstract

A prospective longitudinal cohort study aimed to longitudinally examine the kinetics of anti-Müllerian hormone (AMH) during the first two trimesters of pregnancy. Pregnant women with stored first-trimester serum samples were recruited at 24–28 weeks gestation during their gestational diabetes testing, where they provided an additional serum sample. The samples were analysed for AMH, oestradiol and progesterone concentrations. A decrease in serum AMH was observed in 40 out of 45 (88.9%) (95% CI: 75.9–96.3%) of the participants in this study. The median serum AMH concentration was 10.9 pmol/L in the first trimester and 6.5 pmol/L during the second trimester, with a significantly different distribution of the values between the first-trimester and the second-trimester AMH samples (*P* < 0.001). The median percentage of AMH difference of −39.8%. This study demonstrated a significant decrease in serum AMH levels from the first to the second trimester of pregnancy. The absolute decrease in AMH levels seems to be positively associated with first-trimester AMH levels, whereas the percentage of AMH difference is not. Further studies are required to elucidate the potential physiological mechanisms of this finding.

**Lay summary:**

Anti-Müllerian hormone, also known as AMH, is produced by developing ovarian follicles in the ovary. The concentration of AMH in the serum is used as a marker of ovarian reserve. This marker has been shown to vary throughout the menstrual cycle and in women using hormonal contraception. This study examined this marker in women in the first and second trimesters of pregnancy to determine if it is variable throughout pregnancy. The study found that there was a significant decrease from the first to second trimester, with a larger decrease seen in women who had a higher first-trimester concentration of this marker. Further research is required to determine the physiological mechanism which causes the reduction of AMH in pregnancy.

## Introduction

Anti−müllerian hormone, also known as AMH, is produced by small antral and pre-antral follicles. It is secreted into the follicular fluid and into the circulation, where it can be measured (Durlinger *et al.* 2002*a*,*b*). It has been shown to inhibit excess recruitment of primordial follicles by reducing responsiveness to follicle-stimulating hormone (FSH), thereby selecting the dominant follicle (Peluso *et al.* 2014).

As AMH in the female is only produced by developing pre-antral and antral follicles, it has been postulated that it serves as a marker of ovarian activity. Further, serum AMH has been shown to correlate with the size of the ovarian follicular pool, leading to its widespread adoption as a surrogate marker of ovarian reserve (de Vet *et al.* 2002, Van Rooij *et al.* 2002, Visser *et al.* 2006, Kwee *et al.* 2008).

The serum concentration of AMH was initially thought to remain stable throughout the menstrual cycle (Hehenkamp *et al.* 2006, La Marca *et al.* 2006, Tsepelidis *et al.* 2007); however, recent research suggests that serum AMH can have significant fluctuations throughout the menstrual cycle with an intracycle variation of up to 20.7% (La Marca *et al.* 2009, Dewailly *et al.* 2014, Gnoth *et al.* 2015, Lambert-Messerlian *et al.* 2016, Moolhuijsen & Visser 2020). AMH has also been shown to be impacted by hormonal contraception (HC), with HC use longer than 6 months associated with a decline in serum AMH with recovery after discontinuation of HC (Amer *et al.* 2020).

A recent systematic review demonstrated an association between reduced serum AMH concentration and advancing gestational age, with a post-partum return to pre-pregnancy AMH levels (McCredie *et al.* 2017). However, the majority of evidence regarding AMH in pregnancy is based on cross-sectional studies (La Marca *et al.* 2005, Li *et al.* 2010, Plante *et al.* 2010, Santillan *et al.* 2012, Stegmann *et al.* 2015), with only a few studies longitudinally examining serum AMH fluctuations in early pregnancy (Nelson *et al.* 2010, Koninger *et al.* 2013, Pankhurst *et al.* 2016, Freeman *et al.* 2018), eliminating the problems of significant inter-individual variability in serum AMH seen in cross-sectional studies (Overbeek *et al.* 2012, La Marca *et al.* 2013). As such, the objective of this study is to describe the kinetics of AMH during pregnancy by comparing two measurements from the same patient during the first and second trimesters of pregnancy.

## Materials and methods

### Study design

A prospective longitudinal cohort study was carried out between September 2016 and November 2020 at the Royal Hospital for Women (RHW), Randwick, Australia. The study was approved by the SESLHD Human Research Ethics Committee (Ref number 16-113) and written informed consent was provided by each participant.

### Study population

Women were considered eligible for this study if they carried a singleton pregnancy, were scheduled to undergo a routine oral glucose tolerance test (OGTT) at 24–28 weeks of gestation, at the South Eastern Area Laboratory services (SEALS), and had a stored first-trimester sample available at the RHW from 11 to 13 weeks and 6 days of gestation in this pregnancy. This stored sample would have been obtained as part of the routine first-trimester antenatal screening for chromosomal abnormalities of the fetus, and it is standard procedure at the RHW for this serum to be frozen and stored for up to 18 months. Women were excluded if they had known polycystic ovarian syndrome (PCOS), diabetes or multiple pregnancies.

All pregnant women are routinely screened for gestational diabetes with OGTT at 24–28 weeks of gestation. Women with stored first-trimester samples and upcoming OGTT appointments at SEALS were identified and screened to determine eligibility. Eligible women were approached, and after signed informed consent was obtained, they were recruited for this study. Subsequently, these women were also asked to provide demographic and medical information through a questionnaire.

### Data collection

During the blood collection for the OGTT, an additional 2 mL of blood was drawn into a separate tube. Serum was separated using a Hettich EBA 20 centrifuge at 2404 ***g*** for 10 min and stored at −20°C with its paired first-trimester serum sample, to be analysed at a later stage.

The samples were analysed for AMH, oestradiol and progesterone concentrations, using a Roche e411 analyser. This analyser, with the AMH plus assay, has a lower limit of detection (LLD) of 0.071 pmol/L for AMH, and repeatability and intermediate precision of ≤1.8 and ≤4.4% CV respectively. The LLD of oestradiol is 18.4 pmol/L and 0.159 nmol/L for progesterone.

Demographic and pregnancy-related medical data about the subjects were retrieved using a questionnaire and from their hospital medical file, which contained details of antenatal appointments and previous admissions.

### Sample size

**Figure 1:**
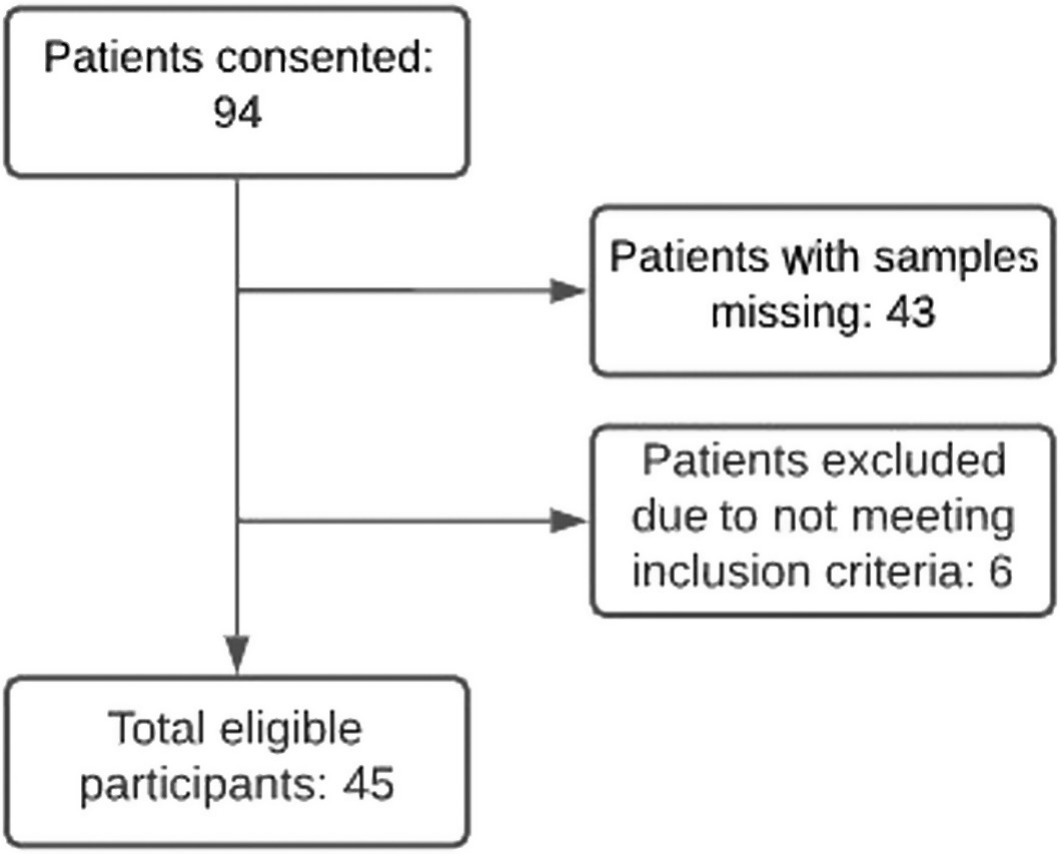
Recruitment flowchart.

Originally a sample size of 100 patients had been calculated for this study to be sufficiently powered to detect a mean difference between the two measurements of 2.5pmol/L with an s.d. of this difference equal to 8 when using a paired Student's *t*-test and an alpha = 0.05 and a beta = 0.10. However, the study was terminated after 5 years of recruitment with a total sample size of 45, due to slow recruitment, as fewer patients were opting for first-trimester antenatal serum screening given the availability of non-invasive prenatal testing. Additionally, the final sample size available for analysis was impacted by the loss of biological samples in the laboratory during the storage period (Fig. 1).

### Statistical analysis

Descriptive statistics were used to provide information about the distribution of the values in the variables assessed. Measures of central tendency (including mean or median) and scatter (s.d. or interquartile range (IQR)) were calculated depending on the normality of the distribution of each variable.

Differences between outcomes were calculated, with the absolute difference in outcome levels defined as second-trimester level minus first-trimester level, where a negative value indicated a decrease in the value from first to second trimester. The percentage of outcome difference was defined as the absolute outcome difference divided by first-trimester level, multiplied by 100.

The comparison of the two AMH measurements for each subject was performed using a Wilcoxon matched-pairs signed-rank test. The association of age, body mass index (BMI), weight, gestational age, first- and second-trimester serum AMH, oestradiol, and progesterone with the observed AMH difference was explored using a Spearman’s rank correlation. A Wilcoxon rank-sum test was then used to assess the difference in absolute AMH decrease and percentage AMH difference by categorical demographic variables including fetal gender, cycle regularity, and use of assisted conception. Furthermore, regression analysis was used to evaluate the association of first-trimester AMH and gestational age with the AMH difference and percentage of AMH difference, whilst controlling for important covariates. STATA 17.0 (StataCorp LP) statistical software was used for this analysis.

## Results

### Participant demographics

The pre-pregnancy characteristics of the included population are presented in Table 1. The age of participants ranged from 25 to 39 years with a median of 34.5 years. The menstrual cycle length in this cohort ranged from 18 to 32 days, with a median of 28 days and menstrual cycles were regular in 75.6% of women. HC had been used in the preceding 12 months by 13 women, whilst 2 women had used assisted conception for this pregnancy.

**Table 1:** Pre-pregnancy characteristics of the participants.

	Median (range)*	IQR	Frequency	%
Age (years)	34.5 (25–39)	31.8–36.9		
Time to pregnancy (months)	1 (0–24)	0.5–4.0		
Average menstrual cycle length (days)	28 (18–90)	27 - 27		
Previous pregnancies^1^	1 (0–6)			
None			13	
1 previous			17	
≥2 previous			12	
Previous births^1^	1 (0–4)			
None			16	
1 previous			23	
≥2 previous			3	
Previous miscarriages^1^	1 (0–5)			
None			34	
1 previous			7	
≥2 previous			1	
Previous terminations^1^	0 (0–3)			
None			38	
1 previous			2	
≥2 previous			2	
Menstrual cycle^2^				
Regular			31	68.9
Irregular			10	22.2
Hormonal contraception use in the prior 12 months^2^				
No			28	62.2
Yes			13	28.9
History of infertility^2^				
No			38	84.4
Yes			3	6.7
Assisted conception^1^				
No			40	88.9
Yes			2	4.4
Smoking^1^				
No			41	91.1
Yes			1	2.2
Alcohol consumption^1^				
No			40	88.9
Yes			2	4.4
Use of illicit drugs^1^				
No			42	93.3
Yes			0	0

^*^Range is minimum to maximum; ^1^relevant information not provided by three patients; ^2^relevant information not provided by four patients.

IQR, interquartile range.

Apart from one woman who had hypothyroidism under treatment, one woman who had a history of endometriosis with previous ovarian surgery and one woman who had experienced antepartum bleeding, this was a healthy cohort with no presence of preeclampsia, gestational hypertension, kidney disease, autoimmune disease or history of chemotherapy or radiotherapy of the pelvis prior to their second-trimester sampling.

### Longitudinal assessment of serum hormonal levels during pregnancy

Two serum measurements were performed during each woman’s pregnancy. The first at a median of 84 days gestational age (range: 77–97 days) and the second at a median of 191 days gestational age (range: 171–205 days).

#### Kinetics of AMH

**Figure 2:**
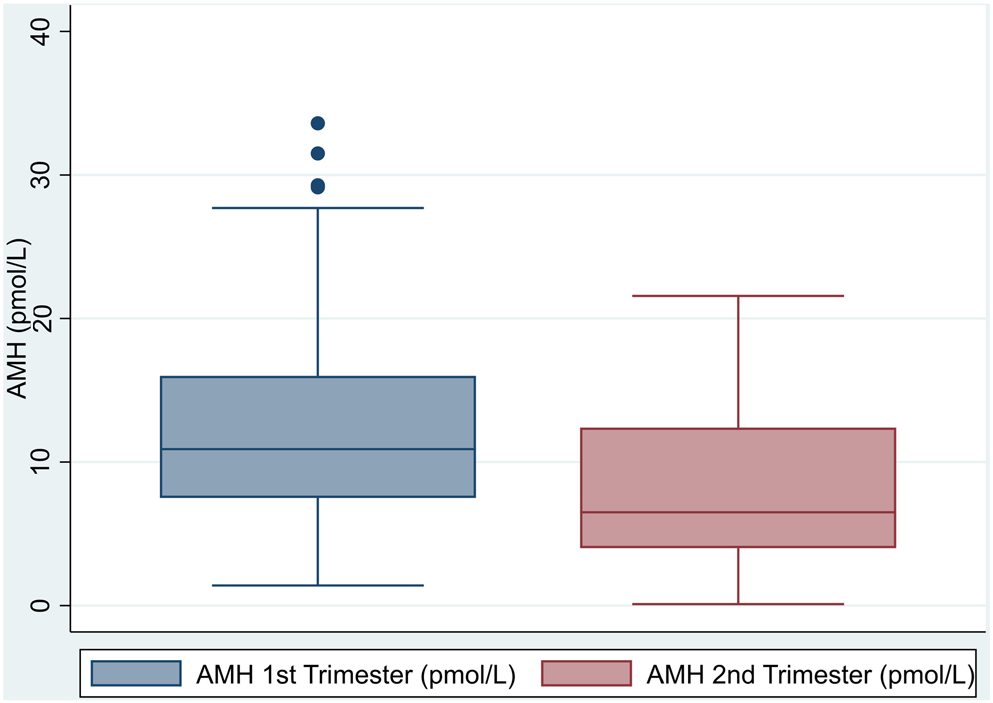
Box plot of AMH levels in first and second trimesters.

The median first-trimester serum AMH concentration was 10.9 pmol/L, while this median was 6.5 pmol/L during the second trimester (Fig. 2).

**Figure 3:**
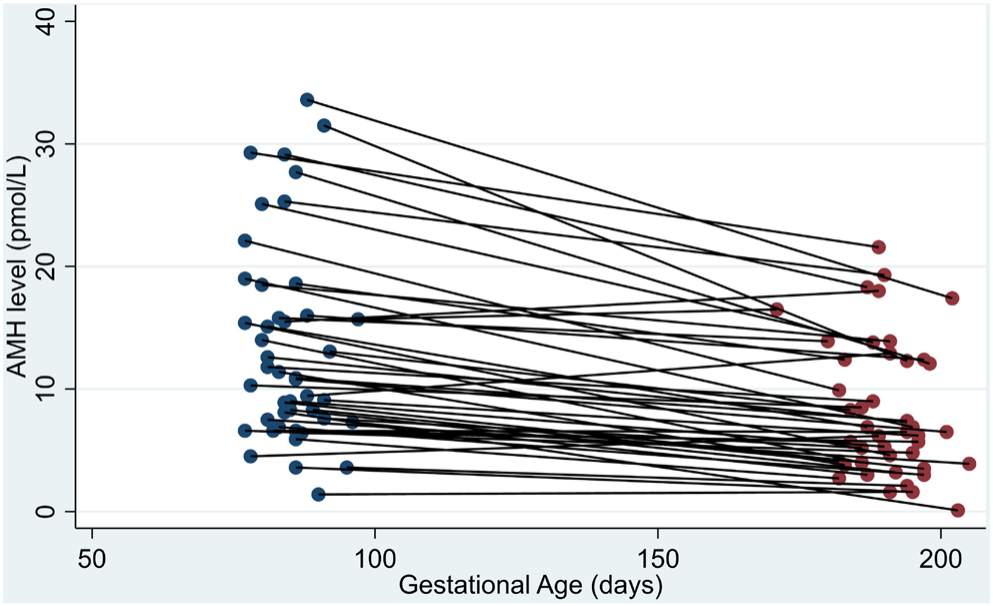
AMH levels by gestational age.

**Figure 4:**
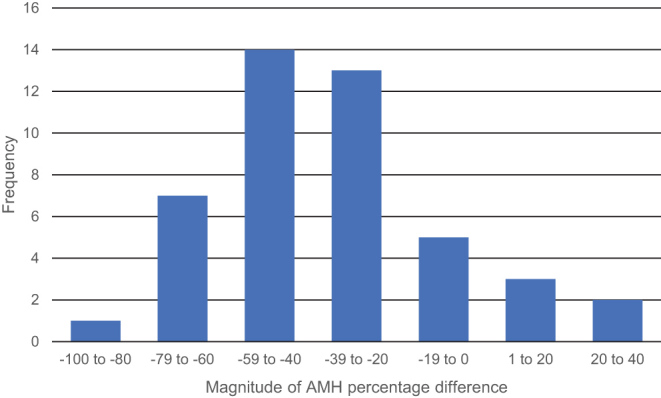
Distribution of percentage of AMH difference.

A decrease in serum AMH was observed in 40 out of 45 (88.9%) (95% confidence interval (CI) 75.9–96.3%) of the participants in this study (Fig. 3). The median of the differences between the first- and second-trimester AMH concentration was −4.2 pmol/L. The median of the percentage of AMH difference was −39.8%. The distribution of the values between the first- and the second-trimester AMH samples was significantly different (*P* < 0.001). The distribution of AMH percentage difference is displayed in Fig. 4.

#### Kinetics of oestradiol and progesterone

The kinetics of oestradiol and progesterone between first and second trimester are presented in Table 2.

**Table 2:** Hormonal monitoring during pregnancy.

	*n*	Range	Median^*^	IQR
Gestational age at first serum measurement, during first trimester (days)	45	77–97	84	81–88
Gestational age at second serum measurement, during second trimester (days)	45	171–205	191	186–195
Difference in gestational age between first and second trimester (days)	45	74–124	107	101–112
AMH first trimester (pmol/L)	45	1.4–33.6	10.9	7.5–16.0
AMH second trimester (pmol/L)	45	0.1–21.57	6.5	4.0–12.4
AMH difference (pmol/L)	45	−19.2 to 3.47	−4.2	−7.71 to −1.9
Percentage of AMH difference (%)	45	−98.77 to 36.81	−39.81	−55.6 to −23.7
Oestradiol first trimester (pmol/L)	38	3553–16,925	7886	6431 to 9251
Oestradiol second trimester (pmol/L)	38	5630–87,490	44,745	31,195–52,285
Oestradiol difference (pmol/L)	38	62–73,240	35,557	24,808–44,214
Percentage of oestradiol difference (%)	38	1.11–830.93	439.08	313.58–583.60
Progesterone first trimester (nmol/L)	37	48.7–217.8	92.6	73.9–125.5
Progesterone second trimester (nmol/L)	37	162.5–474.2	219	183.0–263.1
Progesterone difference (nmol/L)	37	28.5–290.1	125.2	95.85–160.0
Percentage of progesterone difference (%)	37	0–336.70	133.20	89.96–226.24

*A negative value signals a decrease in the level from the first to second trimester.

IQR, interquartile range.

### Predictors of the magnitude of AMH difference

When evaluated with a Wilcoxon rank-sum test, there was no statistically significant association of mean absolute AMH difference or mean percentage AMH difference, respectively, with fetal gender (*P* = 0.78 and *P* = 0.92), cycle regularity (*P* = 0.94 and *P* = 0.15), use of HC in the preceding 12 months (*P* = 0.36 and *P* = 0.48), use of assisted contraception (*P* = 0.13 and *P* = 0.68) and history of infertility (*P* = 0.26 and *P* = 0.52).

#### Absolute AMH difference

The absolute AMH difference (pmol/L) was found to be significantly associated with the first-trimester AMH measurement (r_s_ = −0.67, *P* < 0.001), first-trimester weight (r_s_ = 0.48, *P* < 0.003) and second-trimester weight (r_s_ = 0.43, *P* < 0.005). No other statistically significant associations were observed (Table 3).

**Table 3:** Spearman correlations of AMH difference (second trimester − first trimester) with other variables.

	*n*	AMH difference (pmol/L)		AMH % difference	
		Spearman’s rho	*P* value	Spearman’s rho	*P* value
Age (years)	45	0.09	0.550	−0.04	0.773
First-trimester AMH level (pmol/L)	45	−0.67	<0.001	−0.12	0.436
GA at first AMH measurement (days)	45	0.25	0.093	0.13	0.395
GA at second AMH measurement (days)	45	−0.09	0.54	-0.18	0.230
Difference in GA between measurements (days)	45	−0.26	0.12	−0.21	0.161
First-trimester weight (kg)	41	0.48	<0.003	0.27	0.100
Second-trimester weight (kg)	41	0.43	<0.005	0.30	0.053
Weight difference between first and second trimester	37	0.02	0.90	−0.08	0.644
Weight percentage difference (%)	37	−0.25	0.13	−0.15	0.367
First-trimester BMI (kg/m^2^)	41	0.29	0.08	0.22	0.186
Second-trimester BMI (kg/m^2^)	41	0.24	0.14	0.22	0.162
BMI difference between first and second trimester	37	−0.16	0.34	−0.20	0.234
Oestradiol first trimester (pmol/L)	38	−0.12	0.46	0.19	0.251
Oestradiol second trimester (pmol/L)	38	−0.16	0.33	0.09	0.600
Oestradiol difference (pmol/L)†	38	−0.15	0.34	0.04	0.812
Percentage of oestradiol difference (%)*	38	−0.0262	0.88	0.01	0.933
Progesterone first trimester (nmol/L)	37	0.1221	0.47	0.23	0.165
Progesterone second trimester (nmol/L)	37	0.0557	0.74	0.11	0.503
Progesterone difference (nmol/L)†	36	−0.0780	0.65	−0.13	0.464
Percentage of progesterone difference (%)*	36	−0.0489	0.78	−0.15	0.397
Time to pregnancy (months)	38	−0.1034	0.54	0.07	0.682
Previous pregnancies	42	−0.0059	0.97	−0.30	0.054
Previous births	42	0.1300	0.41	−0.11	0.485

*Difference/first trimester value; †Second trimester − first trimester.

GA, gestational age.

A multivariate analysis was performed to control for potential confounding factors including the difference in gestational age, weight difference and second-trimester oestradiol levels, following which the only strong predictors of AMH difference were first-trimester AMH with a coefficient of −0.47 (95% CI −0.62 to −0.32) and the difference in gestational age between serum measurements, with a coefficient of −0.14 (95% CI −0.26 to −0.21).

#### Percentage of AMH difference

None of the baseline characteristics or hormone levels demonstrated a significant correlation with AMH percentage difference. The strongest correlation was observed with second-trimester weight (r_s_ = 0.30, *P* = 0.053); however, this was not statistically significant. There was no significant correlation with any of the other variables tested (Table 3).

When the multivariate analysis was performed to control for difference in gestational age, weight difference and second-trimester oestradiol levels, the only statistically significant predictor of AMH percentage difference was the difference in gestational age with a coefficient of −1.14 (CI −2.09 to −0.19).

## Discussion

This study demonstrates a significant reduction in serum AMH from the first to second trimester of pregnancy, with a decrease in AMH seen in 88.9% of patients. The magnitude of this decrease in AMH was variable but was greater than 20.0% in 77.8% of patients.

The present study represents the second largest study available exploring this question by employing a longitudinal design. The kinetics of AMH between the first and second trimesters of pregnancy have been evaluated by four other longitudinal studies, which also found a decrease in AMH during pregnancy (Nelson *et al.* 2010, Koninger *et al.* 2013, Pankhurst *et al.* 2016, Freeman *et al.* 2018). The median AMH percentage decrease in these studies varied from 13.5 to 35.9% from the first to the second trimester, consistent with our finding of a median 39.8% decrease during this period. One longitudinal study of 30 women took serial AMH measurements at five time points throughout pregnancy demonstrating decreasing AMH with increasing gestation, with the largest decline in the first trimester between 7 and 14 weeks gestation (Freeman *et al.* 2018). This finding may suggest that the initial steep decline in AMH levels had already begun for women in our cohort whose first AMH measurement was taken at 10–13 weeks gestation.

Some studies have longitudinally evaluated serum AMH levels from the first to the third trimester of pregnancy, with AMH levels declining by up to 64.9% over this time, implying that AMH continues to decline into the third trimester (Nelson *et al.* 2010, Villarroel *et al.* 2018, Pankhurst *et al.* 2021). Unfortunately, this could not be ascertained in our study.

This research question has also been evaluated by cross-sectional studies (La Marca *et al.* 2005, Li *et al.* 2010, Plante *et al.* 2010, Santillan *et al.* 2012, Koninger *et al.* 2013, Gerli *et al.* 2015, Stegmann *et al.* 2015), with all but one (La Marca *et al.* 2005) finding a decrease in serum AMH from the first to the second trimester. Cross-sectional studies are inherently limited compared to longitudinal studies, as they cannot account for inter-individual variation and potential confounding factors. For example, significantly different maternal ages between the cohorts for each trimester would confound the results due to the known association between increased age and decreased serum AMH (Dewailly *et al.* 2014). Other potentially confounding factors include BMI, smoking status and history of infertility (La Marca *et al.* 2013, Jaswa *et al.* 2020).

In the current study, we also calculated the absolute AMH difference between first and second trimesters, and we observed that the higher the first-trimester AMH, the more pronounced the absolute decrease. Interestingly, the percentage of AMH decrease was not associated with the initial AMH level. The fact that the absolute but not relative magnitude of AMH decline correlated with the initial AMH level probably reflects a general mathematical rule no matter whether the AMH kinetic throughout pregnancy follows a first, second or higher order kinetics. However, this cannot be elucidated from the current study and further works on it are needed to provide an answer regarding the exact kinetic profile of AMH throughout human pregnancy. The percentage decrease in AMH between first and second trimesters was found to be up to 98.8% which signifies that the difference between the first- and second-trimester concentrations can be substantial. Nonetheless, the IQR of this measure was 28.2%, indicating a significant variability in inter-individual AMH fluctuations, the exact physiological mechanism of which is currently unknown.

It has been previously suggested that the absolute difference in AMH levels between the first and second trimester is dependent on the woman’s age, with one study finding that there was only a significant difference in AMH levels between different trimesters in women ≤ 34 years (Koninger *et al.* 2013). Although this may be compatible with our findings on the effect of baseline AMH on absolute AMH difference (as younger women tend to have higher AMH), we were unable to find an association between age and AMH decrease. In this study, there was no significant difference in the absolute AMH difference in women ≤ 34 years and women > 34 years (*P* = 0.792), which could be due to the relatively older population in our study (median age of 34.5 years with IQR 31.8–36.9).

Nelson *et al.* (2010) found that AMH was negatively associated with measures of peripheral maternal adiposity and early pregnancy BMI. There was indirect evidence in our study in agreement with this finding, with the demonstration of an association between first-trimester weight and AMH difference. None of the other studies has evaluated this association between weight or maternal adiposity and AMH decrease.

One of the proposed theories regarding the physiological origins of AMH reduction during pregnancy is that it may be due to the elevated oestradiol in pregnancy which suppress follicular recruitment. However, this was not confirmed by the findings of this study as there was no association between serum oestradiol or difference in oestradiol and the difference in AMH between first and second trimesters. It is possible that elevated placental steroids, including oestradiol, suppress the pituitary secretion of FSH in pregnancy (Foyouzi *et al.* 2004), which in turn suppresses the development of the small antral follicles with a subsequent reduction in AMH secretion (Vegetti & Alagna 2006, Dewailly *et al.* 2014). As this is an indirect effect via the pituitary, this might explain why a clear correlation between serum oestradiol and AMH was not detected.

Another proposed theory is that the reduced circulating AMH may be consistent with the natural haemodilution which occurs during the second trimester of pregnancy (La Marca *et al.* 2013), with plasma volume increasing by 29% at the end of second trimester and 48% by the late third trimester (Aguree & Gernand 2019). Pankhurst *et al.* (2016) examined this theory by longitudinally examining the haematocrit during pregnancy, which decreased by a mean of 7.6% from the first to the third trimester but was not correlated with the decrease in AMH during the same period.

A strength of this study, apart from its prospective nature and the longitudinal design, is the use of a contemporary automated AMH assay which has been shown to be sensitive and reliable (Gassner & Jung 2014, Anckaert *et al.* 2016). The samples were also taken within a narrow window of 20 days for the first-trimester measurement and 34 days for the second-trimester measurement, therefore limiting the potential variability originating from differences in gestational age. The study population had limited heterogeneity as participants with PCOS or diabetes were excluded from this study, to make the data less susceptible to the effects of extreme outliers (Kim *et al.* 2016, Al Khafaji *et al.* 2017, Teede *et al.* 2019, Verdiesen *et al.* 2021, Bhattacharya *et al.* 2022). As such, these results cannot be generalised to women with PCOS or diabetes.

The main limitation of our study is that the predetermined sample size was not reached due to logistical reasons, nevertheless, the primary research question of determining whether there is an AMH difference between the first and second trimester of pregnancy was still answered, and a statistically significant reduction was demonstrated. A *post hoc* power analysis revealed that our study’s sample size would have a statistical power of 53.6% to detect the originally hypothesised difference. However, for the observed AMH difference (mean 5.15 pmol/L, s.d. 4.96 pmol/L), the statistical power was 100%, which is why a statistically significant result was still achieved. Nonetheless, the reduced sample size may have limited the ability to perform subgroup analyses, such as when stratifying by age, presenting a risk of type II error for those calculations. Additionally, some patients did not provide data regarding some of the demographic variables which may have affected our ability to demonstrate statistically significant associations between AMH levels and certain demographic parameters.

The findings of the current study suggest areas for further research to identify the potential physiological mechanisms by which the AMH reduction between first and second trimester of pregnancy occurs. A decrease in the rate of follicular recruitment during pregnancy has been hypothesised (Koninger *et al.* 2013), which is consistent with histological evidence of reduced follicular maturation in pregnancy (Govan 1970) and with the findings by some epidemiological studies that increased parity is associated with a delay in the onset of menopause (Whelan *et al.* 1990, Gold *et al.* 2001). Although, no specific details on the exact underlying mechanism are known, if an effect of pregnancy on the rate of follicular recruitment were to be confirmed, and the physiological mechanism underlying this determined, this may lead to the development of interventions to slow the rate of follicular recruitment outside of pregnancy.

An important clinical implication of the current study is that serum AMH in pregnancy should not be used as a predictor of ovarian reserve, especially using nomograms developed with data from non-pregnant patients.

In conclusion, this prospective longitudinal study demonstrated a significant decrease in serum AMH levels from the first to the second trimester of pregnancy. The absolute decrease in AMH levels seems to be positively associated with first-trimester AMH levels, whereas the percentage of AMH difference is not. Further studies are required to elucidate the potential physiological mechanisms of this finding.

## Declaration of interest

There is no conflict of interest that could be perceived as prejudicing the impartiality of the research reported.

## Funding

This work was supported by the School of Women’s and Children’s Health, UNSW.

## Author contribution statement

SM and CV contributed to the design and implementation of the research, to the analysis of the results and to the writing of the manuscript. BA contributed to participant recruitment, data collection and storage or biological samples. MM contributed to study design, storage and analysis of biological samples. WL and CV supervised the study. All authors discussed the results and contributed to the final version of the manuscript.
